# Work after mental-health-related absence: a qualitative study of perceived change after a combination of metacognitive therapy and work-focused interventions

**DOI:** 10.1186/s12889-022-14378-0

**Published:** 2022-11-30

**Authors:** Marianne Tranberg Bjørndal, Fay Giæver, Bente Marianne Aschim, Ragne Gunnarsdatter Hole Gjengedal, Hilde Dallavara Lending, Bente Bull-Hansen, Marit Hannisdal, Odin Hjemdal

**Affiliations:** 1 Division of Mental Health and Substance Abuse, Diakonhjemmet Hospital, Postboks 23 Vinderen, NO-0319 Oslo, Norway; 2 Department of Psychology, Norwegian University of Science and Technology, NO-7491 Trondheim, Norway

**Keywords:** Return to work, Sick leave, Common mental health disorders, Qualitative study

## Abstract

**Background:**

Sick leave caused by common mental health disorders (CMD) is becoming more prevalent. For most people, work is essential for good mental and physical health. It is necessary to provide treatments that facilitate return to work (RTW) and a reduction of symptoms. A qualitative study can contribute to an understanding of what makes an intervention successful. The aim of this study was to investigate how individuals who are on sick leave because of CMD perceive and handle their symptoms and their work, after completing metacognitive therapy and work-focused interventions.

**Methods:**

Semi-structured interviews were conducted with 23 participants after they had completed therapy. Thematic analysis was used to analyse the data.

**Results:**

Through both therapy and the process of RTW, the participants had gained increased awareness and understanding of their mental health problems and the relationship between those problems and work. Together with the sense that they were in charge of their own process of RTW, this helped to improve their self-confidence. An important part of the process was the change to new strategies and the rejection of older maladaptive ones, in relation to both mental health and work. Being open about their mental illness in the workplace could lead to support but also to the opposite, and therefore not an option for everyone. After treatment, most had returned to work and gained a more positive outlook on the future, but some had less confidence in their ability to deal with future symptoms and workplace issues.

**Conclusions:**

Achieving improved self-confidence and adopting new strategies, which enabled them to change how they related to their mental problems and how they addressed their problems at work, seemed to have increased their self-efficacy. Active involvement in therapy and at work was also important, both for the process and as a way of increasing self-efficacy. This gave them renewed belief in themselves and in their ability to handle their work at present and in the future. Despite this being a manualized treatment, the participants’ experience was that it was adapted to each individual, something they regarded as important.

## Background

Work promotes health by providing social interaction, a sense of worth and financial security. Research shows that, for most people, work is essential for good mental and physical health. The benefits of employment include an increased feeling of autonomy, self-reported well-being, and opportunities for personal development [1, 2]. When compared to unemployment, work is associated with fewer symptoms of depression and anxiety [2].

Common mental health disorders (CMD), such as anxiety and depression, affect 20% of the working population at any given time and sick leave due to CMD is increasing [3]. Many people that suffer from CMD receive disability benefits [4]. This is a serious public-health problem and a significant burden for those affected. A study found that individuals on long-term sick leave had negative experiences associated with inactivity, isolation, and changes of self-image, which in turn could lead to experiences of exclusion and stigma [5]. For the employer, this means great losses in productivity, as workers with CMD are more absent from work than others and can have problems performing their work tasks [3]. Sick leave also represents a major challenge for society, as it leads to large costs within social- and health-care systems [3, 6].

The health-care system has traditionally aimed to treat psychological illness by reducing symptoms. Addressing workplace issues and related problems has typically not been its focus [3]. However, there is a growing recognition of the need to develop various measures and initiatives where work is part of the treatment. In a Norwegian context, where the current study is situated, the absence rate is relatively high, and musculoskeletal and mental health problems constitute 60% of sickness absence [7]. It has therefore been seen as crucial to make sure that employees to some extent remain part of the workforce during treatment. Contact with the workplace opens up opportunities for clinical interventions and homework tasks with work-related relevance. This is especially true for individuals suffering from CMD because they often need help with both reducing their symptoms and returning to their workplace [3, 8].

Cognitive behavioural therapy (CBT) shows good results in the treatment of anxiety and depression [9]. However, research indicates that CBT on its own does not reduce work absence, and suggests that there need to be treatments which specifically address work and aid return to work (RTW) [10]. Studies from Germany and The Netherlands have examined the effectiveness of integrating work interventions with CBT. Regular CBT was compared with work-focused CBT for individuals on sick leave because of CMD. These studies found that the treatment had a good effect on symptom reduction and sick leave, but that work-focused CBT was more effective in reducing the number of days of sick leave [11–13]. An observational study from Norway found that a significant larger number of patients in an intervention group returned to work compared to those on a waiting list. The patients were offered either metacognitive therapy (MCT) or CBT and work-focused interventions [14]. These studies therefore support the idea that integrating RTW interventions with treatment has a positive effect on RTW after sick leave. The present study focuses on how patients experience MCT and work interventions as an integrated part of psychological treatment for CMD. MCT is a relatively new treatment for depression and anxiety [15]. The results are promising and possibly even superior to those of other treatment methods [16]. This is also the case in terms of relapse rates [17, 18]. Some early indications from a three-year follow-up study suggest that MCT alone can improve RTW [17]. However, more research is clearly needed.

MCT states that psychopathology is maintained by Cognitive Attentional Syndrome (CAS) which comprises worry, rumination, threat monitoring and maladaptive coping strategies. This CAS activity is governed by metacognitions, which can be both positive and negative. The positive metacognitions are related to the advantages of CAS activity, while the negative ones are mainly related to the uncontrollability of the mental processes in the CAS and the harm they may cause [19]. The treatment focused on challenging beliefs in the negative and positive metacognitions and reducing related CAS activity. The work interventions focused on collecting anamnestic information; exploring work function and potential barriers to RTW, such as bullying and conflicts; informing the patient about the advantages of work; designing an RTW plan; checking whether adjustments in the work situation were needed; and discussing a communication plan with the workplace [20].

The overall goal of the treatment was to reduce symptoms, enhance the patients’ work function, and facilitate their RTW. By interviewing patients who had undergone the treatment, we aimed to capture their experience. Often the patients’ ways of dealing with symptoms and work prior to treatment might not have promoted adaptation, so the treatment involved trying other strategies. A belief that one can effectively use these new strategies is central in the context of treatment. An individual’s intentions and expectations of future work ability have been found to be a predictor for actual RTW [21, 22]. Collectively, such beliefs are often called self-efficacy and refers to individuals’ belief in their own capability to perform certain actions or behaviours effectively [23]. Helping patients to handle their mental health and work may in turn increase their self-efficacy. A relatively new construct, return-to-work self–efficacy (RTW-SE), refers specifically to patients’ belief in their own ability to RTW and handle both their symptoms and work tasks [24]. Having low self-efficacy in relation to work could mean that individuals have a low level of belief that they will be able to RTW and fulfil their work demands or work role [24]. Hence, specifically addressing work within therapy may add the level of specificity required for an improved sense of self-efficacy in a work context. RTW-SE has also been shown to be a strong predictor for RTW for patients suffering from CMD [25, 26].

A recent meta-analysis [27], suggests that sick leave due to CMD often results in low self-efficacy. This analysis found that many employees felt insecure about their ability to handle work demands when returning, and questioned their ability to change their behaviour and personal attributes. Low self-efficacy caused the employees to lose trust in their ability to return to their existing job or get a new one. This might prolong their sickness absence [27].

Finally, the literature often explores and evaluates RTW quantitatively [28], which means that there are few qualitative studies. Qualitative studies can contribute to in-depth knowledge of the RTW process for CMD patients by examining the complex dimensions and practices of RTW [27, 28]. For instance, a qualitative study can show whether, how and why treatment increases self-efficacy, and how this influences the RTW process for the participants. We argue that this study can contribute to an understanding of the qualities that make an intervention successful by examining the factors that employees perceive as facilitators of, and barriers to, the process of RTW. To our knowledge, there have not been any qualitative studies of treatments combining MCT and work focus. Hence, the aim of this study was to explore the experiences of patients who had received the treatment. Our study was guided by the following research question:

*How do participants perceive and handle symptoms and work, following metacognitive therapy and work-focused interventions?*

## Methods

### Study context and design

The current study was conducted in a Norwegian setting. In Norway paid sick leave is granted by a doctor, typically a general practitioner (GP), and employees who are members of the National Insurance Scheme (Folketrygden) are entitled to sickness benefits for a maximum of 52 weeks. If you are absent from work longer than this, 26 weeks without sickness benefits or work allowance, must pass to receive sickness benefits again [29]. Diagnosis, mental or somatic, are confidential, and details about the illness is not revealed to the patient’s employer unless he or she decides to be open about the condition at work. The GP is responsible for the first assessment and when needed will refer patients to specialized health care.

The current study is a qualitative study which was carried out in the context of a randomized controlled trial (RCT) (N = 240) (Clinical Trials NCT03301922, 04/10/2017) conducted at Diakonhjemmet Hospital, Oslo, Norway, as part of “The Norwegian studies of psychological treatment and work” project (NOR-WORK). The inclusion criteria for patients in the clinical trial group was that they were employed, but on sick leave, full or partial, due to depression and anxiety disorders. The patients had been referred to the clinic by their GP and were all above the age of 18. The GP is also typically in charge of prescribing medications and assessing somatic health. Hence, cooperation between the GP and mental health practitioners is usually seen as an integral part of the treatment.

The treatment was offered at an outpatient mental health clinic which is part of the specialised health care system. Treatment for patients in the trial group consisted of a combination of MCT and work-focused interventions. The main focus of the RCT overall was to investigate the effect that treatment had on work status and symptom reduction. Effects were assessed at the end of treatment and at six and twelve months later. The treatment was provided by ten certified metacognitive therapists, of whom nine were psychologists and one was a mental health care worker. The RTW interventions were a set of interventions conducted during the treatment. Assessing the patient’s level of occupational functioning was part of the interventions. Given the patients’ level of functioning and their workplace, individually adapted solutions such as adjustment of work tasks were discussed. The workplace was not actively involved. Involvement of the workplace in regards to disclosure of information and possible adjustments were discussed in treatment and then it was up to the patients how they wanted to involve the workplace. Further detail about the RCT and the treatment can be found in the protocol by Sandin et al. [20].

The current study has a qualitative research design and supplemented quantitative data already collected through questionnaires in the research project. The aim was to contribute to a better understanding of the quantitative data through allowing patients to reflect on, and present, their personal stories and experiences after treatment through in-depth interviews.

### Participant selection/Data collection

The participants were recruited from the RCT. Those who completed their treatment between January and July 2020 were asked to participate in a semi-structured interview. Their therapist gave information about the study, and the patients could opt out of being contacted by telling their therapist. The first author, MTB, made the initial contacts to all the patients, gave further information and invited them to participate. 27 people were contacted, 25 of whom agreed to participate. Of these, one cancelled due to illness and was not available later, while another decided not to participate at the time of the interview. The sample consisted of 5 males and 18 females, who were between 26 and 58 years old. At the start of the treatment all participants were employed but on full or partial sick leave. Some returned to work during treatment and some were still on sick leave, full or partial, after treatment (Table 1). All, except one, remained at the same workplace. Several of the participants adjusted their amount of sick leave during the course of treatment but had some difficulty recollecting the exact times and percentage of their sick leave. A wide range of professions were included in our sample.

**Table 1:** Background information at time of interview

**Gender**	
Female	18
Male	5
**Age**	
26–30	4
31–35	5
35–40	7
41–45	3
46–50	1
51–55	2
56–60	1
**Work status**	
Working	18
Partial sick leave	5
Full sick leave	0
**Education**	
Upper secondary school	4
Higher education 1–4 years	8
Higher education ≥ 4 years	11

### Ethics

The study was approved by the Regional Ethics Committee for Medical and Health Research Ethics in mid-Norway (REK no. 2018/2152) and the Data Protection Office at Diakonhjemmet Hospital. We initially planned to conduct all the interviews face-to-face, but due to the Coronavirus pandemic, 21 out of the 23 interviews were conducted virtually or by phone. The software “Confrere”, a secure video conference system, was utilised for the video interviews. We left it up to the participants to choose whether they preferred video or phone interviews, which meant that eight were conducted by video and 13 by phone. All the participants were given verbal information about the study and gave verbal consent before their interview started. Their written consent form was mailed to them and returned to the researchers. The participants were informed that they could choose not to answer all the questions and could withdraw from the study at any time without consequences. All were given a phone number which they could use to contact the interviewer at a later stage if they had any questions. None of the participants received any compensation for taking part in the study.

### Interviews/Interview guide

The aim of the interviews was to enable the participants to reflect on their own experience of being on sick leave, their treatment and the process of RTW. By asking open questions, we allowed the participants to go deeper into the various topics and issues that came up [30]. An interview guide was prepared and the main topics in the interview were; (1) their reason for seeking treatment; (2) their experience of being on sick leave and the process of RTW; (3) their experience of the treatment; and (4) their cooperation with their GP and others. At the end of the interview, all the participants were asked if there was anything that we had not covered that had been important during their process of returning to work. The interview guide was used to make sure all the topics were covered during the interview.

The interview guide was prepared and revised several times, based on input from the research group and user representatives, and after conducting two test interviews with two clinicians who acted as participants. The interview guide was also discussed and modified again after the first two interviews with actual participants. Specifically, we wanted to know more about their work situations and adjustments made at work, so we tried to have an extra focus on this and questions that allowed them to reflect on this topic. The interviews were conducted from two to four months after the end of the treatment. All the interviews were carried out by the first author, and the third author participated in eight interviews. The interviews lasted between 35 and 90 min, and were audiotaped.

After approximately 15 interviews, we reached saturation and no new information was retrieved. However, we still conducted interviews with all the participants who had agreed to take part and completed their treatment in the time period we had planned for.

All the participants were willing to share their experience of the treatment and their RTW process, and reflect on them. The participants were encouraged to tell their stories. Many talked freely about them, using descriptions and examples, but others talked less freely and needed more follow-up questions. Some became very emotional when telling their stories, but all were comfortable with participating in the study. They were all very grateful for the help they had received and for some it seemed as though their participation in the study was a token of this. The interviewers aimed to be friendly, interested and empathetic, so that the participants would feel comfortable and safe. The first and last questions in the interviews were designed to be more neutral. At the end, the participants were encouraged to ask any questions they might have had. In addition, they were reminded that they could contact the researcher or the clinic at a later stage if they had any questions about the research or needed booster sessions.

### Data analysis

The interviews were transcribed verbatim by the first and third authors and by a student assistant. Identifiable information, such as names, places of work or other personal information that was mentioned, were removed from the transcripts. Thematic analysis [31] was used to analyse the data. This is a flexible analytical tool that can be used relatively independently of theoretical frameworks and epistemological viewpoints. The data was examined for recurring themes that sum up the content, focusing on what was said rather than how it was said [32]. The analytical process consists of six steps: (1) getting to know the data by repeatedly reading the material; (2) initial coding; (3) searching for themes; (4) reviewing themes; (5) defining and naming the final themes; and (6) summarising the analysed material in a final report. The process of analysis is not linear, but one that shifts back and forth between different steps of the analytical process [31]. We started by reading the transcripts several times, and extracting those parts of the interviews which were related to the research question. These were then coded. Based on the different codes, preliminary themes were produced. The interviews and codes were discussed by the different authors, and the themes were adjusted. It was a process where we discussed codes and themes, and went back to the interviews several times, checking that we had found the same themes and had the same understanding of the codes and themes.

## Results

The aim of the study was to investigate the following research question: *How do participants perceive and handle symptoms and work, following metacognitive therapy and work-focused interventions?* First we present themes related to ways in which the participants had changed their perception of their mental health and their situation and the context of this change. Secondly, we present themes addressing the ways in which the participants changed the way they handled their work and their mental illness.

### Change in perceptions of mental health and work

#### Increased awareness and a new understanding of their situation

We found that most participants stated that they had reached a new understanding of their mental health problems. They had gained an increased awareness of the different ways in which their depression and anxiety affected them both physically and mentally in their personal life and at work. Several participants explained how, prior to therapy, they sought treatment for somatic symptoms, but later learned that these symptoms were related to their anxiety and depression, rather than being a somatic illness. In the quotation below, the participant worried that her symptoms indicated a serious underlying somatic disorder such as multiple sclerosis.

I was so tired, I had never experienced this, having physical symptoms, I had headaches every day, I was numb, my legs were tingling, my lips were numb and other strange things so I got very scared (…) I had started thinking that I might have multiple sclerosis or a brain tumour or something, that is why I went to see the general practitioner.

In therapy, they also learned how their worry and rumination were affecting their cognitive abilities, such as through reduced concentration, which resulted in difficulties doing their work or some parts of their work. Initially, many described how their anxiety and depression were affecting them with several negative consequences before they received therapy. They described situations where they felt that they had lost control over their lives and that they were not functioning at work. Below, two participants explained how this was affecting them in different ways:

If you have obsessive thoughts and anxiety of making mistakes at work as a nurse then you kind of have to do something about it before you can go back. I got a very physical reaction from the anxiety, like I almost collapsed at work.

I do feel a lot of sadness, loss of function; having a feeling of losing myself. I was not functioning. I do not have any experience with this from before so it was a very scary situation to be in, and the sick leave was like a confirmation; You are not well enough to go to work.

Some participants described how they initially felt ashamed about their situation. They had never imagined themselves struggling with mental health problems and being on sick leave. Some were worried that they would lose their mind as a result of all the strange thoughts they were having. However, meeting an empathetic therapist, who validated their emotions and feelings and gave support, not only contributed to a better understanding of their symptoms, but also to a reduction of their shame. They realised that depression and anxiety were common, and that others suffering from these illnesses often experienced the same mental processes, such as worry and rumination, and that the coping strategies that they had used before treatment maintained rather than reduced their mental health problems.

#### Improved self-confidence helped to get ready for work

We found that becoming more aware of their worry and rumination, and learning how to relate to these processes differently, created greater understanding. This contributed to a form of security, and gave the participants the feeling of regaining control of their thought processes and ultimately of their lives. The quotation below illustrates how one participant experienced reduced work functioning, and therefore believed that he had ruined his ability to concentrate and then also his ability to perform his work. As he learned to use the new techniques, his worry and rumination were reduced, and he then regained his concentration. This experience was important to him, increasing his self-confidence and self-belief as well as his ability to handle similar symptoms and challenges at work in the future.

It took a long time, and then suddenly I managed it one way or the other and found the concentration I needed, and then I had already started wondering if I had ruined my ability to concentrate, and yes, it is a lot about mastery, I believe in this process for me, to understand that you can, and then suddenly the thoughts calm down and then the concentration comes.

We found that several participants emphasised, in different ways, that they felt in charge of their own processes, and that this was another important way of increasing self-confidence. One participant explained:

I could gradually return to work, feeling that I was in control – now that I was ready for it. And then I felt very ready, and I went to work and it went well.

As mentioned in the quote, they felt in charge of when they should RTW, but also of what happened in the therapy itself.

He (the therapist) took things very gently, so I always felt that it was on my terms. However, he made suggestions and asked questions like: What do you think about this or is this something you would consider trying, after he had justified an exercise.

Being given different techniques and exercises that they could try out, feeling that the treatment was adjusted to them and their needs, and being able to do things at their own pace all helped them to get ready to RTW. Understanding the reasons for, and meaning of, the therapeutic exercises and interventions also gave them an insight into how their mental information processing worked which made the tasks more meaningful.

Even though they stated that they felt in charge of the process, several participants also emphasised that they felt pushed by their therapist, in a positive way, for example to try various exercises at work in between sessions. As one participant mentioned:

“Things that I found uncomfortable, I got pushed to do it anyway”.

This made them try things that they had thought they were not ready for, which often gave them positive experiences.

### Handling work and psychological symptoms in a new way

#### Learning new strategies to handle work

During treatment, the participants became aware of how they reacted, and worked on changing how they related to their thoughts and thinking. One example of this was not engaging with thoughts that triggered ruminations. By doing this, they realised that the strategies they had been using had acted to maintain their problems. One participant explained how she realised this:

I thought maybe that my difficulties were related to the fact that I needed to control my thoughts and what was happening within me and if I were to let go, I would lose control. (…) I was afraid of my own head and afraid of what would happen if I did not stay in control. It was a huge step at the start of the treatment to dare to test out other things, and get a concrete proof of what I had thought the whole time was wrong. That it was not more control I should strive for, but maybe a more relaxed attitude to my inner self.

In the quotation, the participant states that she was trying to control her thoughts, and that trying to do this made things worse for her. When she discovered that she did not have to try to control her thinking but could choose whether to engage in her thoughts or not, then everything changed. For the majority, becoming aware of this was an eye-opener that enabled them to handle and relate to things both at home and work differently and then also reducing their symptoms and not taking up so much of their time.

Overall it was actually about just letting things be. Not ponder on things. If there is a thought that is strange or you think is scary then you should just leave it alone rather than worry about it. And if there are things I cannot do anything about, then there is no point in worrying about them. Also, then, it is actually just to leave it alone, not try to push it away, but just let it hang there. And that was something completely new. It was the opposite of what I had tried for the last 20 years.

Also, participants explained how they learned that monitoring and engaging with their very critical inner voice maintained their mental health problems which then took up a lot of their energy and capacity to for example conduct their work. Being able to refrain from the perseverative thinking process like worry gave them more energy.

I realised that I used a lot of time criticising myself, and I had not really thought about that. So, when I managed to stop with that, then I had a lot of energy left. That was really the turning point.

During the treatment, the participants were encouraged to increase their presence at work and try out their new strategies there. Many considered their work important, valuing its meaning and seeing it as providing a place of belonging. For some, the workplace was a positive place or a space where they could focus on work rather than their problems. Having increased their insight into mental health problems, some realised that avoiding work and, for example, avoiding meetings, was not good for them. If they were to get better, they had to learn how to handle it.

Another possible strategy to better handle work was making adjustments at work. As a result of reduced work function, participants were not able to work as fast or at the same level as they had before. Because of this, and also if they were partly on sick leave, they needed to adjust their work situation, for example by only doing certain tasks. Some noted the importance of doing things they enjoyed or found motivating, and that they needed to change their work routines and their attitude towards their work.

Specific things like before you leave work you should make a plan for tomorrow, focusing on completing one thing before you start another, and that kind of specific rules, that we made together, are very useful, so I have written it down, so when I start my computer, they are in front of me.

#### Being open about mental illness at work

We found that there were large variations in the participants’ attitudes towards openness about mental problems at work. However, they had all considered how to relate to this issue. Several participants stated that they were selective about the kind of information they disclosed and to whom. Also, some noted that they chose to share information with people who would give them support and recognition about their problems. However, for some, being open at work was not an option. This could have been because of the type of work or the work situation. It was also noted that it would be easier to talk freely about struggling with something else, rather than with a mental illness. A couple of participants described not being met with understanding when talking about their symptoms and that this was a painful experience.

That is probably typical, those who do not give support, take more focus than those who give support, I notice that it makes me feel a bit betrayed. It is complicated at a workplace, talking about it, it is of course a lot easier to break a leg, than to say that you have mental problems, but I stood for it facing most people at least, but it is exhausting to have it, it would have been easier to be home because of something else.

However, the majority of the participants who had been open about their mental illness at work said that they had been received in an accepting and empathetic way, and that this support and understanding was important when returning to work. Sharing information about their mental health facilitated an interaction with their supervisor or colleagues that made returning to work easier for them. For some, openness led to understanding from the supervisor, and this resulted in a temporary reduction of demands and pressure at work which facilitated the RTW process. One participant, who stated that she told her supervisor everything, said:

The most important thing was actually to go to her and tell her what was working for me or not. And that she understood why, and that I did not have to come to work and pretend that everything was fine when it didn’t really feel like that. It was one thing less to worry about, that I didn’t have to conceal how things were.

#### Handling future problems at work

At the time of the interviews, all the participants stated that they were doing better, and were feeling stronger and more secure in the ways in which they related to their problems, regardless of whether they had returned fully to work or not. Many felt that they had completely recovered and had a very positive outlook on the future. Some participants who had been struggling with anxiety attacks noted that they had stopped having them, while others explained how they had stopped ruminating and worrying. This resulted in a completely different life.

I handle things in a very different way. Have more energy, and I just feel that it has helped me a lot. In a short amount of time really.

Other participants explained how they were doing better but still struggling with different types of symptoms. They were glad to have fewer problems but also described that they had accepted that they had anxiety symptoms, and were now less worried about it. An important aspect was that they believed that they would recognise maladaptive thought processes earlier, and therefore, would be able to act sooner and handle future problems better. One participant explained:

Most importantly for me is that I know what to do and I know that it works so if I get worse again I know I have the tool, and that is a very big relief in itself which reduces the worries.

As in the quotation above, the participants in general described a more positive outlook on the future. Increasing their awareness and understanding of their own mental-health issues and learning new techniques and strategies made them feel more secure about themselves and gave them the sense that they would be able to handle potential future problems in a better way than before. However, a couple of participants, who were still struggling, were concerned about future problems and their ability to use what they had learned during the treatment to handle their work and their symptoms.

In a way I have instructions regarding how I can handle this, and I try to use them when I have these negative thoughts, I try to use the tool. But it is not very easy so I do not know, I do not know if I can handle it on my own.

## Discussion

The results show that the participants have, in different ways, changed how they perceive and handle their work and mental illness as a result of the treatment. During the process of returning to work, they had different experiences that contributed to this change, which seemed overall to have increased their self-efficacy and given them a more positive outlook on RTW and on the future in general. In the current study, changing to different strategies seemed to build the participants’ belief in their ability to deal effectively with their own thought processes. This may again have contributed to boosting their self-efficacy in relation to their mental health, RTW and handling work tasks once again. They realised that their constant worry and rumination was taking up a lot of their time and mental capacity, making it difficult for them to do other tasks. Being in charge of their RTW process and making good decisions for themselves also seemed to be important ways of gaining a feeling of self-confidence, and thereby increasing their self-efficacy.

Central to MCT is tackling maladaptive repetitive thought processes such as worry and rumination. A very interesting aspect of the results was the way in which the participants’ worries related to their reduced functioning, and how this might have been indications of serious somatic symptoms. By becoming aware of the thought processes and strategies that maintain psychopathology, many of the participants had been able to change the ways in which they related to them, and how they acted. This change also affected many of the symptoms of concentration problems, reduced energy, headaches and feelings of numbness, which the participants initially feared were caused by somatic conditions. This seemed to have restored their confidence that they would once again be able to handle their mental health problems and work, even though some of them were still struggling with anxiety and depression.

At the time of the interviews, all participants stated that the treatment had helped them, although in different ways. Some felt that they were better but not completely recovered, while a minority were still struggling and were not sure how to handle their problems or how to use what they had learnt in the future. These participants described how the mental processes of worry and rumination still remained to different extents, and said that they were still affecting their level of functioning. The participants described changes in their declarative knowledge about ways of thinking and relating to their thoughts. However, the lack of procedural descriptions of their different actions might indicate that the participants found it difficult to translate their new knowledge into practice. This, in turn, could negatively influence the individual’s self-efficacy, in relation both to handling mental health and returning to work. This could potentially be a reason for prolonged sickness absence as found in Andersen and colleagues [27]. The treatment in the current study was relatively short, so some participants might have needed more time to benefit fully from it. More time could have given the participants more experience of using their new strategies. It is also possible that these participants might have benefited from regular booster sessions for a longer period after treatment, something which has been suggested in other studies and could be investigated further [33].

There could be different reasons why some were still struggling and had not returned to work. Two participants had experienced bullying and harassment at work and had not returned for this reason. Previous research has shown that patients exposed to bullying report significantly higher levels of depressive and anxiety symptoms, as well as lower levels of self-efficacy, job satisfaction and have higher prevalence of sick leave [34]. These patients may need more time to return fully to work, as doing so often involves finding a new workplace, which normally may require more time. Furthermore, different types of work interventions such as individual placement and support have been found useful to aid RTW for people suffering from more severe mental health illness and in need of more supported employment [35, 36]. Patients that RTW later could also be struggling with other unresolved issues either at work or at home [37–39].

The treatment ended either just before or during the first four months of the Coronavirus pandemic. This may have had negative implications for RTW of some of the participants. Having the opportunity to try out new strategies at work may also be an important intervention during MCT and work focus, and staying at home would have made this difficult. Working from home may feel positive in the short term for participants struggling with anxiety and depression, and could also be used as a means for adjustments when getting ready to RTW, but for some it may also overlap with the maladaptive coping strategy of avoidance. In such cases, working from home could potentially maintain and increase mental health problems over time. The reduced social contact may contribute to higher levels of isolation, which could also enhance a feeling of hopelessness and other depressive symptoms.

Consistent with other recent findings [40], this study found that a common dilemma was whether or not to inform the employer about one’s mental illness and if so, how much should be disclosed. In Norway, information about the health issue which has led to sick leave is confidential, and so sharing it depends on what the employee wants to disclose. Sharing could both lead to support but can also be experienced negatively as seen in Noordik and associates [41]. This is in line with the findings of this study, where participants describe similar experiences of disclosing information about mental-health problems at work. Some had been open and some had not because of feared negative response. In general, it seemed as that the participants in the current study had carefully considered who they talked to and shared information with. This could be seen as an important strategy in the RTW process. Findings by Brouwers and colleagues [40] highlights the potential of a strategic disclosure process in regards to who to disclose to, timing and what is being said as a promising measure for improving work participation and decreasing discrimination and stigma. Another Dutch study [42] showed that the majority of the workers would disclose a mental illness to their managers. Important aspects were their relationship with the manager and the previous experience of colleagues. But the study also found that a significant proportion would not disclose because they feared negative consequences. In a study by Coleman and colleagues [43] one such feared negative consequence was that disclosure would damage future career opportunities. In our study, those participants who had disclosed information about their illness were mostly met with supportive and empathetic responses. Their openness also made it possible for their employers to adjust their work situations. However, several participants in our study had decided not to share because they did not believe they would receive an accepting response. This underlines that being open about mental health problems is highly contingent on an individual’s circumstances and work environment. If being open leads to support it can be beneficial, but there is no guarantee of this. Negative experiences can on the other hand be very difficult to handle. Therefore, it is important first to explore whether the employee’s work environment is conducive to sharing. A work environment involving bullying would, for instance, be an exception to this. For those that choose to share it is not uncommon to worry about the perceptions of others, which may increase reluctance and worry. Both sharing and postponing the worrying related to this may have served as a corrective experience for the participants, as there is a discrepancy between worrying about a negative outcome and their managers response.

Getting treatment can in itself be thought of as an active choice. This treatment programme required participants to actively participate in the treatment both during sessions and between them. All the participants stated that they wanted to RTW. Moreover, tackling workplace problems, such as the need for supervisors and colleagues to be given information and make adjustments, could be seen as producing active changes in their situation. This made the participants take greater charge of their own situations which, especially when the experience was positive, contributed to an increase in their self-efficacy in relation to tackling their mental health problems and their work. Having experience of testing new strategies at work or with work-related content may be essential for a successful RTW process. When it comes to the employer it can be argued that it is more important for them to know something about the current level of functioning and capacity of the employee, hence the diagnosis is not of relevance. Focusing on what the employee can and cannot do at the moment and making temporary changes in the work situation could contribute in their RTW process. So, when being on sick leave and asking for adjustments, the focus could be specifically addressing this and not their diagnosis. This could in turn promote useful adjustments of work tasks. Focusing less on symptoms and more on capabilities is well established within the International Classification of Functioning, Disability and Health (ICF) concept developed by the World Health Organization [44]. The participants described their experiences of being in control of their own processes, feeling met by the therapist, and having the interventions focused on their situation. This indicates that even when they received a protocol-based therapy, they sensed that it was tailored to them as individuals, which optimized the relevance of the therapy. The therapy was overall structured as a set of sequences focusing on mental processes and work-related interventions. The content of the processes was based on what each participant described in an initial case conceptualization, which summarised the patient’s symptoms and the work-related problems. This may be why participants described the treatment interventions as being personalized to their individual needs, and this may also have facilitated both their symptomatic recovery and their functional recovery related to their mental-health problems and work situation. As a result, this may have increased their self-efficacy in relation to both their symptoms and their work.

### Implications for mental health practitioners

Based on the findings in this study, we argue that there are some important implications for mental health practitioners, particularly pointing to barriers and facilitators for successful RTW interventions.

Barriers may potentially relate to practical issues, such as patients not being able to dedicate time and effort to therapy or the RTW process due to other commitments. It is also worth noting that the treatment requires motivation and dedication to practice in between sessions and that drop out may become an issue. This highlights the importance of the therapist being attentive to and addressing motivation during therapy. It is also crucial for the therapist to be sensitive to, and to address potential stigma related to mental health issues in therapy to reduce feelings of shame so that the patient remains committed to participate fully, and to try out various strategies in real life in between sessions. Another issue to be aware of is the length of treatment. Some of the informants in our study felt that the intervention did not last long enough and experienced a sudden lack of support after the intervention was ended. One way of addressing this issue could be through offering booster sessions aimed at repeating and adjusting insights and strategies from the intervention. Mental health practitioners should also be mindful of work-related conflicts, such as bullying and harassment. In these cases, it may be particularly difficult, or not even advisable, for the patient to return to that particular workplace. In cases where the issue cannot be resolved, it may be more fruitful to encourage the patient to explore alternative options for employment.

There were several facilitators that can be pointed to from the findings of the current study. Among these were the importance of creating motivation for treatment. Being met by the therapist and feeling pushed in a good way, to try out what they had learned, was important for the motivation. Practicing between session by e.g. doing homework between sessions is also essential. A solid understanding of the patient’s mental health problems and work situation is needed. This is achieved by a systematic assessment of each individual’s situation. Also, it is particularly important for the participant to be able to increase their awareness of their thought processes, and how this is affecting their mental health and their work functioning.

We also argue that it is important for participants to feel responsible for, and in charge of, their individual process during the intervention and the RTW process. This involves being included and making active choices in regards to treatment and when to increase work hours and finally fully returning to work. In a Norwegian context, this implies a good cooperation with the Norwegian Welfare and Labour Administration (NAV) and especially the GP as they prescribe the sick leave.

Finally, an important facilitator is to perceive the therapy received as relevant for their particular problem and situation. This is possible also for treatments that use a treatment protocol. This is also the case for the current treatment. It is generic and highlight mental processes that are involved in common mental disorders.

### Strengths, limitations and suggestions for future research

Conducting semi-structured interviews gave the participants the possibility of describing in depth their experience of the treatment and the RTW process. The current study selected participants from a defined period during the RCT, and all those who completed their treatment in this period were asked to participate. The sample was heterogeneous in terms of type of work and age. There were more women than men, which also reflected the nature of the participants included in the RCT as a whole. We did not find any gender differences in their experiences of the RTW process, however this was not a focus point.

There was some variation in the length of time that had passed after the completion of the treatment at the time of the interview. This might have affected what they reported in different ways. For example longer time meant for some that they had more time to use what they had learned but for others this passing of time meant they had forgotten their new techniques as they had not continued practicing. We only interviewed participants who had completed the treatment. This might constitute a bias, as these participants might have been more motivated and more satisfied, since they had not dropped out of the treatment. However, we were interested in how the treatment had contributed to their mental health and RTW. The responses of those who had dropped out would therefore not contribute with information which was relevant to this particular research question. Also, we only talked to each participant once. Future studies could strengthen the data by interviewing the patients at several points of time, such as before, during and after the treatment.

## Conclusion

In this study, the participants reported that their perception of their mental illness and work-related problems had changed as they gained an increased awareness that gave them a new understanding of their problems. Another factor which was important for getting ready to RTW was improved self-confidence, which they gained from feeling in charge of their own process, through active involvement in their therapy and the method and timing of their RTW. These aspects were important in increasing the participants’ RTW self-efficacy. By gaining new strategies and losing old maladaptive ones, the participants learned to handle their symptoms and work differently. They described how the mental strategies enabled them to change not only the ways in which they related to their mental problems but also how they addressed their problems at work. This also increased their self-efficacy in relation to their mental health and RTW, giving them renewed belief in themselves and in their ability to handle their work both at the present time and in the future.

## Data Availability

The participants in this study have not consented to distribution of the data outside the study conducted at the clinic and the conditions regarding confidentiality, privacy protection and data handling approved by the Regional Ethics Committee and the Data Protection Office at Diakonhjemmet Hospital. Request can be directed to MTB at Marianne.bjorndal@diakonsyk.no.

## List of abbreviations

**CMD**: Common mental health disorders
**CBT**: Cognitive behavioural therapy
**RTW**: Return to work
**MCT**: Metacognitive therapy
**CAS**: Cognitive attentional syndrome
**RTW-SE**: Return to work self-efficacy
**RCT**: Randomized controlled trial
**GP**: General practitioner
**NOR-WORK**: The Norwegian studies of psychological treatment and work
